# Innovative technique for managing extreme relapsing bilateral pseudoangiomatous stromal hyperplasia (PASH) in a young woman: A case report highlighting a novel intervention in reconstruction

**DOI:** 10.1016/j.ijscr.2024.109873

**Published:** 2024-06-06

**Authors:** Yeganeh Pakbaz, Parisa Hoseinpour, Faranak Olamaeian, Nahid Nafissi

**Affiliations:** a Breast Health & Cancer Research Center, School of Medicine, Iran University of Medical Sciences, Tehran, Iran; b Department of Pathology, Breast Cancer Research Center, Motamed Cancer Institute, ACECR, Tehran, Iran

**Keywords:** Pseudoangiomatous stromal hyperplasia, Benign breast disease, Immediate implantation technique, Skin-reducing mastectomy, Case report

## Abstract

**Introduction:**

Pseudoangiomatous stromal hyperplasia (PASH) is a rare breast stromal lesion that typically manifests clinically as a palpable unilateral, painless lump that is freely movable and has a rubbery or firm consistency. The diagnosis can be confirmed by a core needle biopsy (CNB) or surgical excision. Treatment options include medical treatment with hormonal management for asymptomatic patients or local excision and breast reduction in rare cases.

**Case presentation:**

We reported the case of a 24-year-old woman with a history of taking contraceptive pills for about a year. Examination revealed extremely enlarged, sore, and swollen breasts, particularly the right side, marking her third PASH relapse. The patient underwent a surgical skin-reducing mastectomy (SRM) using a novel technique with an immediate prepectoral implant covered by a dermal flap to reconstruct the breast shape due to the large PASH lesions and aiming for optimal cosmetic outcomes. The procedure was complication-free with no recurrence after 18 months of follow-up.

**Discussion:**

Mastectomy followed by immediate implantation offers benefits such as prompt restoration of breast shape with fewer surgeries.

**Conclusion:**

This case report highlights the successful use of immediate implantation in reconstructing large recurrent benign breast diseases. The outcomes indicate that immediate implantation shows promise as a suitable choice for carefully selected patients managing large, relapsing bilateral benign breast diseases. However, due to common complications such as infection and implant loss, it is not generally recommended for benign lesions. The decision should be made on a case-by-case basis, considering the size, recurrence, and individual suitability.

## Highlights

• PASH typically grows slowly, instances of rapid growth are rare.
• Symptomatic and recurrent cases can benefit from surgical interventions such as breast reduction.
• SRM improves aesthetic outcomes and psychological well-being.
• Mastectomy with immediate reconstruction is promising for managing recurrent huge PASH.

## 1 Introduction

Pseudoangiomatous stromal hyperplasia (PASH), first described by Vuitch et al., is a rare benign proliferative lesion of breast stroma that generally manifests as a palpable, unilateral, painless, movable lump with a rubbery or firm consistency, often diagnosed incidentally through pathological examinations to distinguish it from other benign lesions [[1], [2], [3], [4]]. It typically presents as a mass-forming lesion, a mammographic abnormality, or an incidental histologic finding in the context of fibrocystic change, fibroepithelial lesion, gynecomastia, and, rarely, invasive carcinoma. The etiology of PASH involves hormonally driven proliferation [5].

PASH predominantly affects women of reproductive age, with a mean age of 40 [2]. While PASH lesions are mostly well-defined and small, some cases are large, with the largest reported by Brodie et al. measuring 27 × 22.6 × 7.4 cm [6]. Although PASH typically grows slowly, instances of rapid growth are rare [1].

Diagnostically, PASH often appears as a mass on mammography, usually well-defined without calcifications, or as an asymmetrical density. Ultrasonography typically shows a well-defined mass with low echogenicity or echogenic regions with linear hypoechoic structures [7,8]. The diagnosis is confirmed through a core needle biopsy (CNB) or surgical excision [8,9].

Treatment options for PASH include medical management with hormonal therapy for asymptomatic patients, or surgical interventions such as local excision, partial mastectomy, or breast reduction, for symptomatic cases. Recurrence rates vary widely, from 0 % to 22 % [10]. Recurrent cases often require re-excision or mastectomy, especially in multifocal instances [11,12].

Skin-reducing mastectomy (SRM) is a surgical technique utilized in breast cancer treatment that removes breast tissue while preserving the natural skin envelope. SRM offers aesthetic benefits, particularly for patients with larger breasts or significant ptosis or sagging. It reshapes the breast skin during the mastectomy to achieve a tighter and more youthful contour using autologous dermal flap prepectoral reconstruction and immediate implant placement.

The use of the patient's tissue in creating a natural pocket for the implant contributes to a more natural outcome and better long-term results, enabling the surgeon to achieve better symmetry and contour during the immediate reconstructive phase, ultimately leading to improved patient satisfaction and psychological well-being [13,14].

The key principle of SRM involves meticulous planning and execution to completely remove breast tissue while preserving the native skin envelope. Additionally, autologous dermal flap prepectoral reconstruction, a component of SRM, uses the patient's tissue to create a natural pocket for the implant without the need for biological or synthetic mesh or acellular dermal matrix (ADM) [15]. This innovative approach enhances implant coverage with de-epithelialized skin from the lower part of the breast to cover the entire anterior surface of the implant without cutting any muscle, providing stability and durability of the prepectoral plan, minimizing certain complications, and maintaining a more natural appearance [16].

Furthermore, this approach has demonstrated reduced postoperative pain, foreign body infection, improved muscle function, and a lower risk of implant loss, capsule contracture, and animation deformity. By retaining the skin envelope and optimizing the aesthetic outcome, patients who undergo SRM may experience improved self-esteem and body image following their treatment [17,18]. There is a lack of information regarding the use of mastectomy and immediate breast implant reconstruction for PASH cases [6].

We represent a significant bilateral and progressive case of huge recurrent PASH, highlighting the potential effectiveness of mastectomy and immediate breast reconstruction as a treatment option for this benign tumor. This approach, although typically reserved for malignant breast cancer, shows promise in addressing recurrent large PASH.

## 2 Presentation of case

Following the SCARE guidelines, this clinical case report aims to offer a detailed description of the surgical procedures [19]. A 24-year-old woman presented with a three-month history of painless, significant, and rapidly progressive enlargement of the right breast.

She had a previous medical history of benign breast disease and underwent surgery two times—six months and two years before the current presentation. Initially, she presented with two painless palpable masses in the upper and lower medial areas of the left breast, measuring 5.2 × 4.0 × 7.6 cm and 5.0 × 2.7 × 4.8 cm, respectively. The masses were removed through a partial mastectomy in February 2020, with pathological analysis following a CNB that confirmed PASH. There was no family history of breast disease. At presentation, the patient was not taking any medication but noted she had taken oral contraceptive pills for about a year, which she discontinued two years ago. She experienced menarche at age 13 and had regular menstrual cycles.

**Fig. 1:**
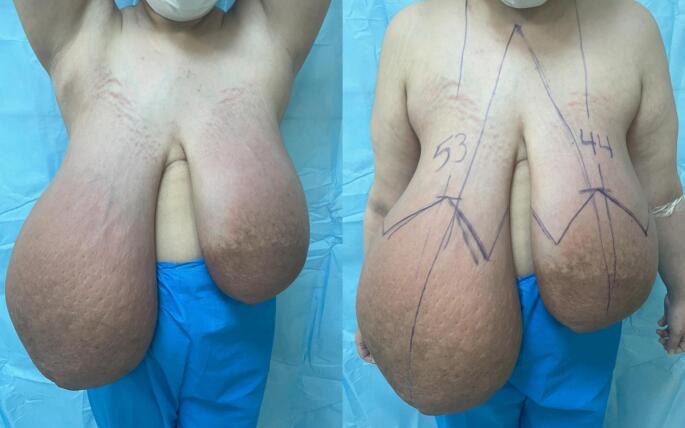
Preoperative image: pre-operative image photographs of a 24-year-old woman presenting with extreme breast enlargement with edema.

On breast examination, we found both breasts, particularly the right, were asymmetrically and extremely enlarged. The skin on both breasts was sore and swollen. A palpable mass in the right breast encompassed the entire breast, estimated to weigh approximately 4–5 kg. Additionally, on the left side, a substantial mass weighing 2–3 kg and occupying over 75 % of the left breast, was palpated (Fig. 1). No axillary or supraclavicular lymphadenopathy was palpated.

**Fig. 2:**
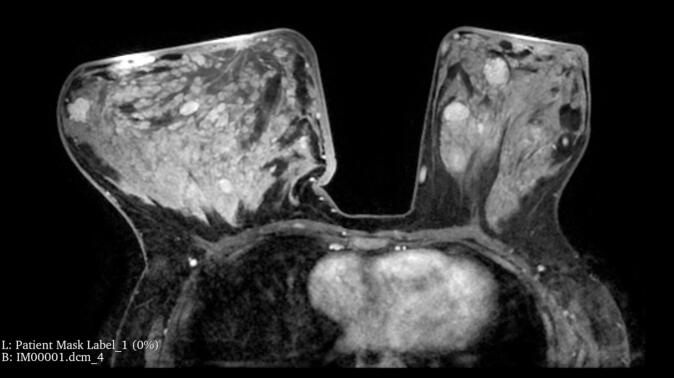
Preoperative radiologic findings: magnetic resonance imaging findings. Severely dense fibroglandular tissue, diffuse parenchymal inflammation with skin thickening, and areolar edema in the right breast.

**Fig. 3:**
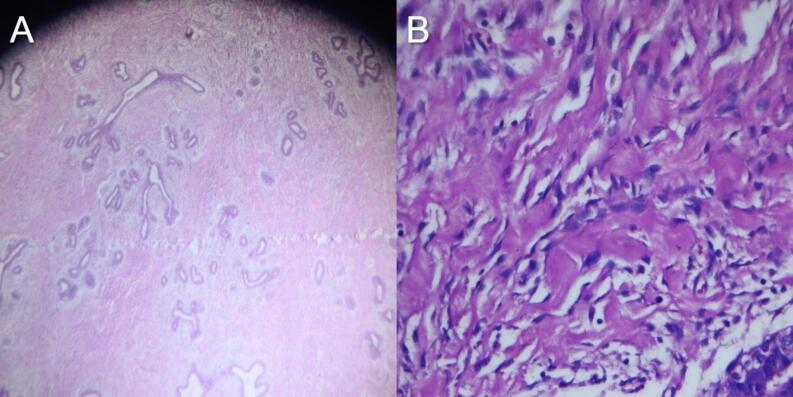
Histopathological analysis: (A) low-power view (H&E, ×40): Histopathological slide showing PASH. (B) High-power view (H&E, ×400): mild proliferation of ducta and lobules nearly collapsed by a massive dense fibrotic stroma is observed.

We requested ultrasonography, core needle biopsy, and magnetic resonance imaging (MRI) as part of the diagnostic workup (Fig. 2). Ultrasound revealed multiple bilateral hypoechoic circumscribed masses, severe edematous changes, and a heterogeneous fibroglandular parenchymal pattern. The largest masses were 5.0 × 1.6 cm in the right retroareolar zone and 6.2 × 2.7 cm in the lateral part of the left retroareolar zone, with no suspicious masses (BIRADS: III). MRI revealed severely dense fibroglandular tissue, diffuse parenchymal inflammation, skin thickening, and areolar edema in the right breast. Pathology showed fibrotic breast tissue with fibroadenomatoid mastopathy, equivocal histopathological features of PASH, and focal inflammation, mainly in subcutaneous areas (Fig. 3).

**Fig. 4:**
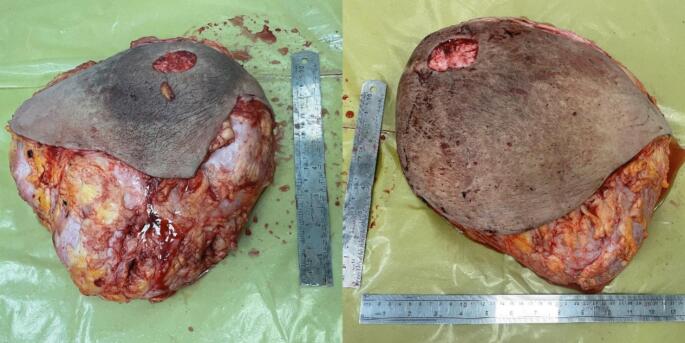
Gross appearance of the specimen: resected bilateral breast specimens.

**Fig. 5:**
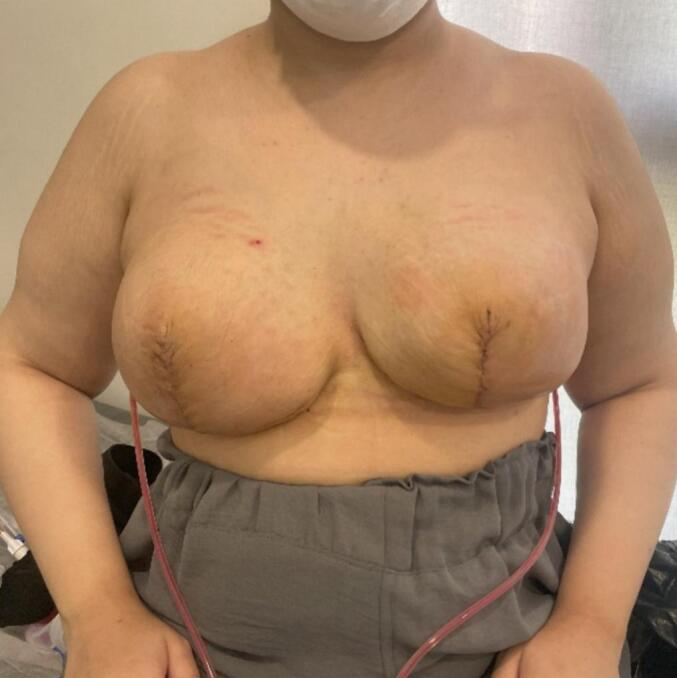
Outcome at the two weeks follow-up: two weeks post-operative photos of the patient.

The patient underwent an SRM with an inferior pedicle technique. Her sternal notch to nipple (SN-N) measurement was 53 cm on the right side and 44 cm on the left side, resulting in a new SN-N measurement of about 22 cm. The inferior flap measured 20 × 30 cm on the right side and 18 × 30 cm on the left side. This flap was de-epithelialized, as in an inferior flap reduction mammoplasty. After detaching all breast tissue from the skin envelope, the lower part of the breast tissue was resected from this large dermal flap, and hemostasis was achieved (Fig. 4). After irrigation with saline and antibiotics, the 500-cm^3^ round implants were placed prepectorally. The inferior dermal flap was then used to completely cover the implant. Subsequently, the skin was closed in an inverted T pattern in three layers. It was decided not to use the NAC (nipple-areolar complex) as a free flap to reduce the risk of recurrence and minimize complications associated with NAC necrosis. The procedure was performed without complications, and the patient was satisfied with the result (Fig. 5). She has been followed for 12 months with no signs of recurrence.

## 3 Discussion

PASH is a rare benign breast condition characterized by abnormal growth of stromal tissue, typically presenting as palpable breast masses or non-palpable radiographic abnormalities [2]. However, in rare cases, PASH cases can be large and bilateral, causing significant anxiety and concern for women, especially when a mass is present [[1], [2], [3], [4]].

Surgical intervention may be necessary for large or symptomatic PASH lesions, ranging from excision or lumpectomy to mastectomy, based on lesion extent and patient preference. In cases of massive and recurrent PASH, mastectomy followed by breast reconstruction can be an option [[10], [11], [12]]. Immediate implantation post-mastectomy restores breast shape and volume. This new method offers benefits such as fewer surgeries and better cosmetic results [20].

While breast reconstruction post-mastectomy can provide significant psychological benefits, it is essential to note the complexity and potential risks and complications, including infection, implant rupture, capsular contracture, skin necrosis, and implant loss. Women considering breast reconstruction should discuss the potential risks and benefits with their surgeons and make informed decisions based on their circumstances.

This case highlights the potential utility of immediate prepectoral implantation with a dermal flap in managing large PASH lesions to achieve optimal cosmetic outcomes and prevent recurrence. However, it is important to note that this technique may not be appropriate for all cases and should be considered on an individual basis.

In conclusion, this case report demonstrates the successful use of immediate implantation with full coverage of the implant by a large dermal flap without using any ADM or synthetic mesh for the reconstruction of recurrent benign breast disease involving the entire breast tissue. A young woman with massive bilateral PASH may be chosen to undergo mastectomy and implantation for a variety of reasons, including symptom relief and improved aesthetic outcomes. However, due to common complications such as infection and implant loss, this procedure is not generally recommended for benign lesion surgery. Therefore, the decision to use immediate implantation should be made on a case-by-case basis, considering factors such as the size and recurrence of the disease, as well as the overall suitability for each patient.

Further studies are needed to evaluate the efficacy and safety of this technique in larger patient groups. The results from this case report suggest that immediate implantation may be a viable option for selected patients with recurrent, extremely large benign breast disease who desire mastectomy.

## Guarantors

Dr. Nahid Nafissi and Dr. Yeganeh Pakbaz.

## Ethical approval

The ethics clearance was not necessary for this study because our institutional review board does not require ethical approval for reporting individual cases or case series.

## Funding

This research did not receive any specific grants from funding agencies in the public, commercial, or not-for-profit sectors.

## Statement of informed consent

Written informed consent was obtained from the patient for publication of this case report and accompanying images. A copy of the written consent is available for review by the Editor-in-Chief of this journal on request.

## Authors' contribution

Yeganeh Pakbaz: Writing – original draft (lead); investigation (lead). Parisa Hoseinpour: Resources (lead). Faranak Olamaeian: Writing – review and editing (equal). Nahid Nafissi: Conceptualization (lead); writing – review and editing (equal); Supervision (lead).

## Declaration of competing interest

There is no conflict of interest between the authors as everybody is aware of the work and participated actively.
